# Supplementary material for: Data-driven identification of biological systems using multi-scale analysis
Source: PLoS Comput Biol. 2025 Nov 6;21(11):e1013193. doi: 10.1371/journal.pcbi.1013193 (PMC12611157; doi:10.1371/journal.pcbi.1013193)
Supplement: S1 Appendix — (PDF) [file pcbi.1013193.s001.pdf]

S1 Appendix. Application to a 3-dim stochastic model with one transition from fast to slow.

The implementation of the proposed framework to a higher dimensional system will be demonstrated here on the basis of the following 3-dim Biofilms model [1]:

$$\begin{aligned}\frac{dG_p}{dt} &= k_1 G_E G_p - k_4 G_p - k_2 A G_p \\ \frac{dA}{dt} &= -k_3 A + k_5 G_i \\ \frac{dG_i}{dt} &= k_4 G_p - k_5 G_i\end{aligned} \tag{1.1}$$

The system is simulated with the parameters values: $k_1 = 0.3426\ \left(\text{mmol}\,\text{L}^{-1}\,\text{s}^{-1}\right)^{-1}$, $k_2 = 5.3\ \left(\text{mmol}\,\text{L}^{-1}\,\text{s}^{-1}\right)^{-1}$, $k_3 = 4\ \text{s}^{-1}$, $k_4 = 2\ \text{s}^{-1}$, $k_5 = 30\ \text{s}^{-1}$, $G_E = 10\ \text{mmol}\,\text{L}^{-1}$.

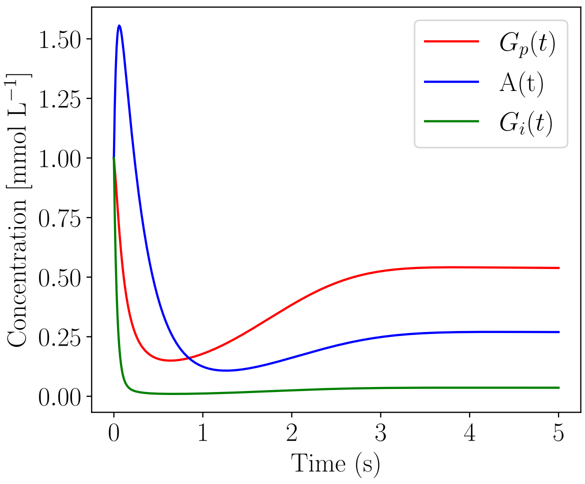

Fig A. System Solution. Temporal variation of the system's variables. ICs: $G_p(0) = 1$ mmol L^{-1}, $A(0) = 1$ mmol L^{-1}, $G_i(0) = 1$ mmol L^{-1}.

The system parameters are chosen so that G_i rapidly transitions to a quasi-steady state, consistent with the assumptions outlined in Garde *et al* [1]. With noise-free full simulation data, Weak SINDy successfully recovered the Full system model. However, with introduction of 2% additive and multiplicative noise, Weak SINDy was unable to recover the full system model. 1 presents the details of the identified models from noise-free simulation data and simulated data with addition of 2% noise .

CSP analysis reveals the presence of a distinct timescale gap in the system, as illustrated in Fig B (left). Following the initial transient dynamics, the intracellular metabolite G_i rapidly relaxes to a quasi-steady state [1], which can be expressed as

$$\frac{dG_i}{dt} = k_4 G_p - k_5 G_i \approx 0. \tag{1.2}$$

From this, the quasi-steady state approximation yields

$$G_i \approx \frac{k_4}{k_5} G_p. \tag{1.3}$$

Substituting Eq. (1.3) into the first two equations of the full biofilm system (Eq. 1.1)

Table 1. Comparison of the right-hand side expressions between the ground truth and the identified models by the Weak SINDy

Noise Type	Ground Truth	Identified model	R^2
Noise-free	$\dot{G}_p = 1.426G_p + 5.3AG_p$	$\dot{G}_p = 1.426G_p + 5.3AG_p$	1.0
	$\dot{A} = -4A + 30G_i$	$\dot{A} = -4A + 30G_i$	1.0
	$\dot{G}_i = 2G_p + 30G_i$	$\dot{G}_i = 2G_p + 30G_i$	1.0
Additive Noise	$\dot{G}_p = 1.426G_p + 5.3AG_p$	$\dot{G}_p = 1.463G_p - 1.708{G_P}^2 - 1.333A^2 - 130.164{G_i}^2$	0.99997
	$\dot{A} = -4A + 30G_i$	$\dot{A} = 1.796G_p - 3.889A0.056{G_p}^2 + 47.388{G_i}^2$	1.0
	$\dot{G}_i = 2G_p + 30G_i$	$\dot{G}_i = 0.629{G_p}^2 - 132.924{G_i}^2$	1.0
Multiplicative Noise	$\dot{G}_p = 1.426G_p + 5.3AG_p$	$\dot{G}_p = 1.469G_p - 2.182{G_p}^2 - 1.382A^2 - 30.702{G_i}^2$	-10.3310
	$\dot{A} = -4A + 30G_i$	$\dot{A} = 1.912G_p - 4.022A + 0.184A^2 + 30.810{G_i}^2$	-0.7264
	$\dot{G}_i = 2G_p + 30G_i$	$\dot{G}_i = 0$	-21.2553

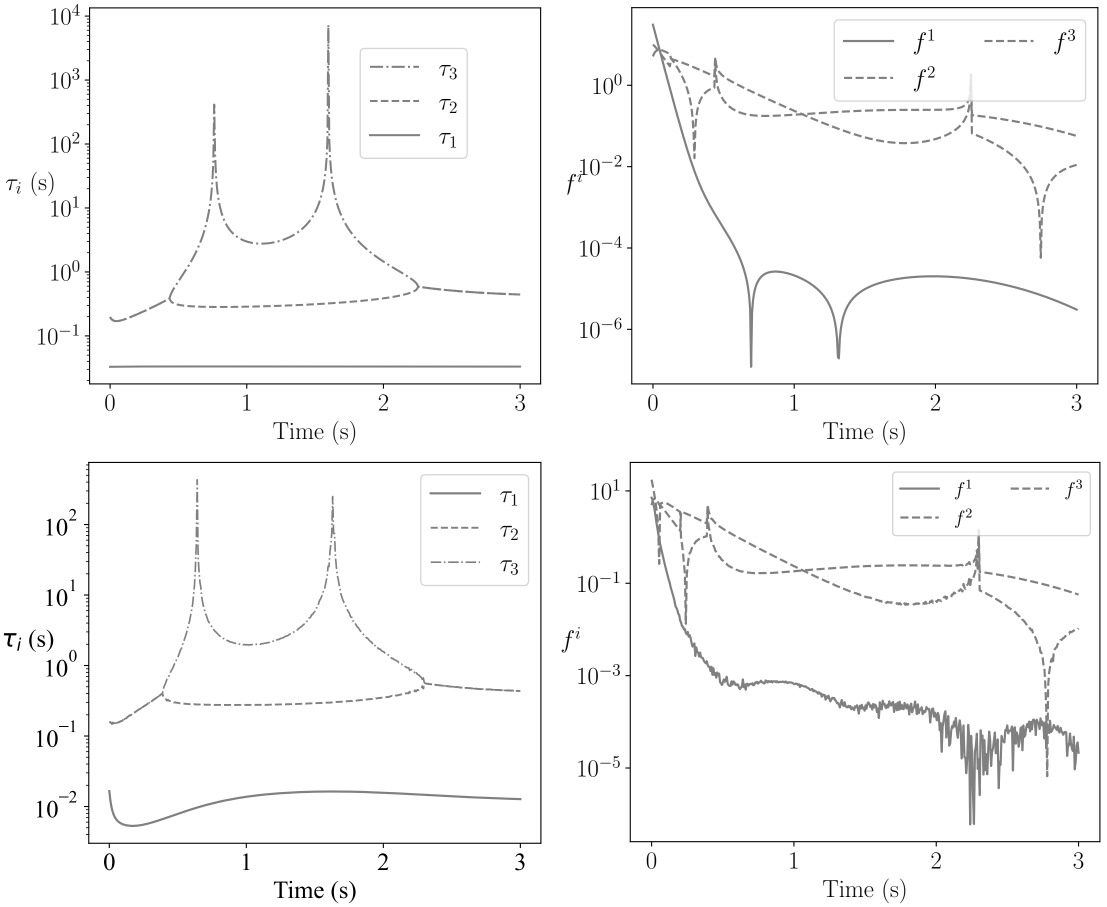

Fig B. CSP Analysis. The evolution in time of the developing time scales (left column), and of the amplitudes of the related CSP modes (right column), evaluated from the ground truth model (top row) and from the proposed framework (bottom row).

leads to the reduced two-dimensional dynamics:

$$\begin{aligned}\frac{dG_p}{dt} &\approx k_1 G_E G_p - k_4 G_p - k_2 A G_p,\\ \frac{dA}{dt} &\approx -k_3 A + k_4 G_p.\end{aligned} \tag{1.4}$$

Taking the derivative of Eq. (1.3) and substituting $\frac{dG_p}{dt}$ from Eq. 1.1, we obtain

$$\frac{dG_i}{dt} \approx \frac{k_4}{k_5} \left(k_1 G_E G_p - k_4 G_p - k_2 A G_p\right). \tag{1.5}$$

Thus, the slow reduced-order system consists of Eqs. (1.4) and (1.5).

Data corresponding to the region in which G_i has relaxed to its quasi–steady state was utilized to train the Weak SINDy algorithm for reduced model identification. To further assess robustness, we introduced 2% additive and multiplicative noise to the dataset and repeated the identification procedure. The resulting models are summarized below.

Table 2. Comparison of the right-hand side expressions between the ground truth and the the identified models by the proposed framework

Noise Type	Ground Truth	Identified model	R^2
Noise-free	$\dot{G}_p = 1.426G_p + 5.3AG_p$	$\dot{G}_p = 1.426G_p + 5.3AG_p$	0.9998
	$\dot{A} = -4A + 2G_p$	$\dot{A} = -3.934A + 1.957G_p$	0.9998
	$\dot{G}_i = -0.35333AG_p + 0.095067G_p$	$\dot{G}_i = 0$	-3.4290
Additive Noise	$\dot{G}_p = 1.426G_p + 5.3AG_p$	$\dot{G}_p = 1.407G_p + 0.112{G_p}^2 - 5.451AG_p$	0.9995
	$\dot{A} = -4A + 2G_p$	$\dot{A} = -4.049A + 2.011G_p$	0.9993
	$\dot{G}_i = -0.35333AG_p + 0.095067G_p$	$\dot{G}_i = 0$	-3.4290
Multiplicative Noise	$\dot{G}_p = 1.426G_p + 5.3AG_p$	$\dot{G}_p = 1.424G_p - 0.037{G_p}^2 - 5.212AG_p$	0.9998
	$\dot{A} = -4A + 2G_p$	$\dot{A} = -3.954A + 1.969G_p$	0.9999
	$\dot{G}_i = -0.35333AG_p + 0.095067G_p$	$\dot{G}_i = 0$	-3.4290

Despite the introduction of both additive and multiplicative noise, the reduced model could still be identified. The additional terms (highlighted in red in Table 2) were insignificant in magnitude compared to the dominant coefficients of the identified models.

References

1. Garde R, Ibrahim B, Kovács ÁT, Schuster S. Differential equation-based minimal model describing metabolic oscillations in Bacillus subtilis biofilms. Royal Society Open Science. 2020;7(2):190810.
