# Supplementary material for: Data-driven identification of biological systems using multi-scale analysis
Source: PLoS Comput Biol. 2025 Nov 6;21(11):e1013193. doi: 10.1371/journal.pcbi.1013193 (PMC12611157; doi:10.1371/journal.pcbi.1013193)
Supplement: S2 Appendix — (PDF) [file pcbi.1013193.s002.pdf]

S2 Appendix. Application to a 3-dim model with two transitions from fast to slow and slow to slower.

The proposed framework is now applied in the case of a 3-dim system which exhibits two dinstict transitions, one from fast to slow and another from slow to much slower. Let's consider the following 3-dim system:

$$\begin{aligned} \dot{y}_1 &= -\frac{1}{\epsilon_1} y_1 + k_3 y_2 + k_2 y_3, \\ \dot{y}_2 &= -\frac{1}{\epsilon_2} y_2 + k_3 y_1 + k_1 y_3, \\ \dot{y}_3 &= -\frac{1}{\epsilon_3} y_3 + k_2 y_1 + k_1 y_2, \end{aligned} \tag{2.1}$$

where k_1, k_2 and k_3 are constants that are set to 1 for simplicity, and $\epsilon_1 = 10^{-4}$, $\epsilon_2 = 10^{-2}$, $\epsilon_3 = 1$. Fig A displays the solution of the system,

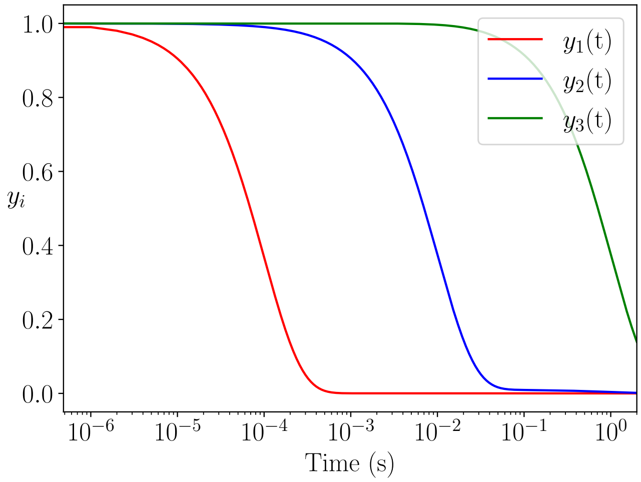

Fig A. System Solution. Temporal variation of the system's variables. ICs: $\boldsymbol{y}_0 = [1, 1, 1]^\top$. x-axis in logarithmic scale.

The parameters of the system are set so that y_1 exhibits the fastest transition towards a quasi-steady-state, followed by y_2, while y_3 undergoes the slowest transition. SINDy/Weak SINDy is unable to identify the underlying dynamics of this system, as its multiscale nature does not allow the construction of a single model valid across the entire timescale spectrum. Therefore, the following analysis is conducted solely on the absense of noise, since SINDy/Weak SINDy fails even in this case. The conclusions are subsequently extended to the stochastic model.

Table 1. Comparison of the right-hand side expressions between the ground truth and the identified model by the Weak SINDy, using the full dataset.

Ground Truth	Identified model	R^2
$\dot{y}_1 = -10000y_1 + y_2 + y_3$	$\dot{y}_1 = -0.01y_2$	1.0
$\dot{y}_2 = -100y_2 + y_1 + y_3$	$\dot{y}_2 = -100y_2 - 0.01y_1 + y_3$	1.0
$\dot{y}_3 = -y_3 + y_1 + y_2$	$\dot{y}_3 = -y_3 + y_2$	1.0

CSP analysis demonstrates that two time scale gaps develop, as shown in Fig B (top-left). A similar behavior is exhibited by the time scales calculated on the basis of

our proposed framework, as shown in Fig B (bottom-left). The related CSP modes' amplitudes for the explicit model are displayed in Fig B (top-right). The amplitudes become exhausted progresively. In particular, f^1 becomes negligible first at around $t_1 = 2 \times 10^{-3}$ s, setting its related mode exhausted. Then f^2 becomes negligible next, setting the second mode exhausted at around $t_2 = 2 \times 10^{-1}$ s, while the amplitude f^3 remains large keeping its related mode active for the period shown. The same behavior is manifested on the basis of our proposed framework. Fig B (bottom-right) shows that f^1 and f^3 become negligible at around the same times.

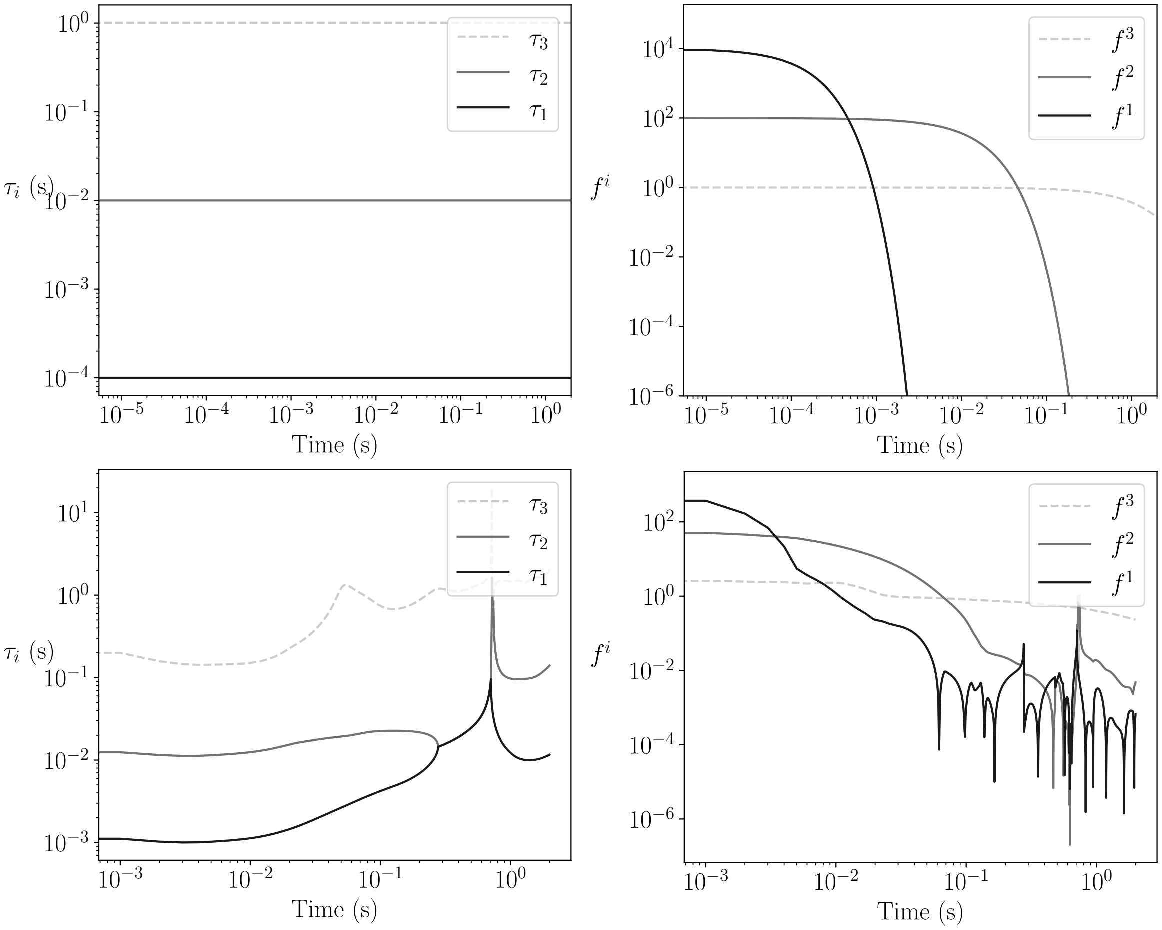

Fig B. CSP Analysis. The evolution in time of the developing time scales (left column), and of the amplitudes of the related CSP modes (right column), evaluated from the ground truth model (top row) and from the proposed framework (bottom row). x-axis in logarithmic scale.

During the initial evolution and up to t_1, the system is described in full by Eq. (2.1) and no reduced model needs to be constructed, as all modes are active. Between t_1 and t_2 the first mode becomes exhausted indicating a quasi-steady-state for variable y_1:

$$\begin{aligned} f^1 \approx 0 &\Rightarrow -\frac{1}{\epsilon_1} y_1 + y_2 + y_3 \approx 0 \\ &\Rightarrow y_1 \approx \epsilon_1 (y_2 + y_3) \\ &\Rightarrow \dot{y}_1 \approx \epsilon_1 (\dot{y}_2 + \dot{y}_3) \end{aligned} \tag{2.2}$$

By substituting $\dot{y}_2$ and $\dot{y}_3$ from Eq. (2.1) we get the description of the valid reduced model in this region:

$$
\begin{aligned}
\dot{y}_1 &\approx 2\epsilon_1 y_1 + \left(\epsilon_1 - \frac{\epsilon_1}{\epsilon_2}\right) y_2 + \left(\epsilon_1 - \frac{\epsilon_1}{\epsilon_3}\right) y_3 \\
\dot{y}_2 &= -\frac{1}{\epsilon_2} y_2 + y_1 + y_3 \\
\dot{y}_3 &= -\frac{1}{\epsilon_3} y_3 + y_1 + y_2
\end{aligned} \tag{2.3}
$$

Substituting the fast variable y_1 from Eq. (2.2) yields:

$$
\begin{aligned}
\dot{y}_1 &\approx \left(2\epsilon_1^2 + \left(\epsilon_1 - \frac{\epsilon_1}{\epsilon_2}\right)\right) y_2 + \left(2\epsilon_1^2 + \left(\epsilon_1 - \frac{\epsilon_1}{\epsilon_3}\right)\right) y_3 &&= -\frac{494999}{50000000} y_2 + \frac{1}{50000000} y_3 &&\approx -0.009899 y_2 \\
\dot{y}_2 &\approx \left(\epsilon_1 - \frac{1}{\epsilon_2}\right) y_2 + (1+\epsilon_1) y_3 &&= -\frac{999999}{10000} y_2 + \frac{10001}{10000} y_3 &&= -99.9999 y_2 + 0.9999 y_3 \\
\dot{y}_3 &\approx (1+\epsilon_1) y_2 + \left(\epsilon_1 - \frac{1}{\epsilon_3}\right) y_3 &&= -\frac{9999}{10000} y_3 + \frac{10001}{10000} y_2 &&= -0.9999 y_3 + 1.0001 y_2
\end{aligned} \tag{2.4}
$$

In a similar manner, when the second mode becomes also exhausted after t_2, a quasi-steady-state is established for both variables y_1 and y_2:

$$
\begin{aligned}
f^1 \approx 0 &\Rightarrow -\frac{1}{\epsilon_1} y_1 + y_2 + y_3 \approx 0 \\
&\Rightarrow y_1 \approx \epsilon_1 (y_2 + y_3) \\
&\Rightarrow \dot{y}_1 \approx \epsilon_1 (\dot{y}_2 + \dot{y}_3) \\
f^2 \approx 0 &\Rightarrow -\frac{1}{\epsilon_2} y_2 + y_1 + y_3 \approx 0 \\
&\Rightarrow y_2 \approx \epsilon_2 (y_1 + y_3) \\
&\Rightarrow \dot{y}_2 \approx \epsilon_2 (\dot{y}_1 + \dot{y}_3)
\end{aligned} \tag{2.5}
$$

By solving for y_1 and y_2 in the RHS of Eqs. (2.5) we conclude:

$$
\begin{aligned}
y_1 &\approx \frac{\epsilon_1(1+\epsilon_2)}{1-\epsilon_1\epsilon_2} y_3 \quad &&\Rightarrow \dot{y}_1 \approx \frac{\epsilon_1(1+\epsilon_2)}{1-\epsilon_1\epsilon_2} \dot{y}_3 \\
y_2 &\approx \frac{\epsilon_2(1+\epsilon_1)}{1-\epsilon_1\epsilon_2} y_3 \quad &&\Rightarrow \dot{y}_2 \approx \frac{\epsilon_2(1+\epsilon_1)}{1-\epsilon_1\epsilon_2} \dot{y}_3
\end{aligned} \tag{2.6}
$$

Substituting $\dot{y}_3$ from Eq. (2.1) we get the description of the valid reduced model in this region:

$$
\begin{aligned}
\dot{y}_1 &\approx \frac{\epsilon_1(1+\epsilon_2)\left(\frac{(1+\epsilon_1)\epsilon_2}{1-\epsilon_1\epsilon_2} + \frac{\epsilon_1(1+\epsilon_2)}{1-\epsilon_1\epsilon_2} - \frac{1}{\epsilon_3}\right)}{1-\epsilon_1\epsilon_2} y_3 &&= -\frac{99979597}{999998000001} y_3 \\
\dot{y}_2 &\approx \frac{(1+\epsilon_1)\epsilon_2\left(\frac{(1+\epsilon_1)\epsilon_2}{1-\epsilon_1\epsilon_2} + \frac{\epsilon_1(1+\epsilon_2)}{1-\epsilon_1\epsilon_2} - \frac{1}{\epsilon_3}\right)}{1-\epsilon_1\epsilon_2} y_3 &&= -\frac{9899959897}{999998000001} y_3 \\
\dot{y}_3 &= \left(\frac{(1+\epsilon_1)\epsilon_2}{1-\epsilon_1\epsilon_2} + \frac{\epsilon_1(1+\epsilon_2)}{1-\epsilon_1\epsilon_2} - \frac{1}{\epsilon_3}\right) y_3 &&= -\frac{989897}{999999} y_3
\end{aligned} \tag{2.7}
$$

It is demonstrated in Table 2 that three different models can be constructed, representative of each of the three different regions of the solution, where different dynamics prevail. This is possible by following the proposed framework; i.e. by

partitioning the solution into the three regions, as proposed by the CSP analysis, our framework was able to identify a valid model in each region. Table 2 presents the details of the identified models after splitting the solution according to CSP.

Table 2. Comparison of the right-hand side expressions between the ground truth and the identified models by the proposed framework.

Region	Ground Truth	Identified model	R^2
Split 1 (Full)	$\dot{y}_1 = -10000y_1 + y_2 + y_3$	$\dot{y}_1 = -10000y_1 + y_2 + y_3 + 0.005y_1(y_3 - 1)$	1.0
	$\dot{y}_2 = -100y_2 + y_1 + y_3$	$\dot{y}_2 = -100y_2 + y_1 + y_3 - 0.226y_1(y_3 - 1)$	1.0
	$\dot{y}_3 = -y_3 + y_1 + y_2$	$\dot{y}_3 = -y_3 + y_1 + y_2 + 0.032y_1(y_3 - 1)$	1.0
Split 2 (QSSA_{y_1})	$\dot{y}_1 = -0.009899y_2$	$\dot{y}_1 = -0.01y_2$	0.99997
	$\dot{y}_2 = -99.9999y_2 + 0.9999y_3$	$\dot{y}_2 = -100y_2 - 0.01y_1 + y_3$	1.0
	$\dot{y}_3 = -0.9999y_3 + 1.0001y_2$	$\dot{y}_3 = -y_3 + y_2$	1.0
Split 3 (QSSA_{y_1,y_2})	$\dot{y}_1 = -0.00009998y_3$	$\dot{y}_1 = 0$	-4.75235
	$\dot{y}_2 = -0.00989998y_3$	$\dot{y}_2 = -0.01y_3$	1.0
	$\dot{y}_3 = -0.989898y_3$	$\dot{y}_3 = -0.99y_3 - 0.01y_2$	1.0

Note that for Split 1, the extra identified terms highlighted in red in 2 are negligible and the identified model simplifies to the ground truth model, since at this region $y_3 = 1$. For Split 2 and Split 3 regions, the extra identified terms highlighted in red are also negligible.
