# Supplementary material for: Data-driven identification of biological systems using multi-scale analysis
Source: PLoS Comput Biol. 2025 Nov 6;21(11):e1013193. doi: 10.1371/journal.pcbi.1013193 (PMC12611157; doi:10.1371/journal.pcbi.1013193)
Supplement: S3 Appendix — (PDF) [file pcbi.1013193.s003.pdf]

S3 Appendix. CSP Methodology.

Assume that we have an N-dim. biological system, the evolution of which is described by the system of ODEs

$$\frac{d\mathbf{y}}{dt} = \mathbf{g}(\mathbf{y}) \tag{3.1}$$

where $\mathbf{y} = [y_1, y_2, ..., y_N]^\top$ and $\mathbf{g}(\mathbf{y}) = \left[\frac{dy_1}{dt}, \frac{dy_2}{dt}, ..., \frac{dy_N}{dt}\right]^\top$ are the N-dim. column state vector and vector field. According to CSP, any multi-scale system in the form of Eq. (3.1) can be cast into a form where the vector field $\mathbf{g}(\mathbf{y})$ is decomposed into N different terms that are defined by the CSP basis vectors $\mathbf{a}_n$ that span the fast and slow subspaces of the tangent space [1, 2]:

$$\frac{d\mathbf{y}}{dt} = \mathbf{g}(\mathbf{y}) = \sum_{n=1}^{N} \mathbf{a}_n f^n, \qquad f^n = \mathbf{b}^n \cdot \mathbf{g}(\mathbf{y}) \tag{3.2}$$

where each $\mathbf{a}_n f^n$ term is called a CSP mode and is a linear combination of all processes and variables of the system. $\mathbf{a}_n = \mathbf{a}_n(\mathbf{y})$ and $\mathbf{b}^n = \mathbf{b}^n(\mathbf{y})$ are the N-dim. CSP column and row, respectively, basis vectors of the n-th mode, and $f^n = f^n(\mathbf{y})$ is the amplitude of the n-th mode. The CSP basis vectors satisfy the orthogonality conditions $\mathbf{b}^i(\mathbf{y}) \cdot \mathbf{a}_j(\mathbf{y}) = \delta^i_j$ [1, 3], and proper adjustment of their signs sets f^n always positive. Each CSP mode describes subprocesses that act in different time scales and have different impact in the vector field. In particular each CSP basis vector $\mathbf{a}_n$ provides the direction along which a subprocess is evolving according to the n-th fastest time scale, and the amplitude f^n measures how much the vector field is projected along the $\mathbf{a}_n$ direction; i.e. it measures the impact of the n-th CSP mode on $\mathbf{g}(\mathbf{y})$. The basis vectors that construct order by order the expressions in Eq. (3.2) are provided by CSP in an algorithmic manner [4, 5].

In the absence of the expolicit expression of the equations of the system in Eq. (3.1), the calculation of the CSP basis vectors is not always possible. Instead, approximations of the CSP basis vectors can be used to identify the fast/slow directions. Assuming that we have a dataset of N variables y_i $(i = 1, ..., N)$, the derivatives $\mathbf{g}(\mathbf{y})$ of which are calculated using finite differences, the Jacobian Matrix $\mathbf{J}$ of the system can be then estimated using the combination of NODE [6] and the NN of [7] described previously. Then, we can use the right, $\boldsymbol{\alpha_n}$, and left, $\boldsymbol{\beta^n}$, eigenvectors of the calculated Jacobian matrix as a leading order approximation of the CSP basis vectors to span the fast and slow subspace of the vector field [1, 2]; $\mathbf{a}_n \approx \boldsymbol{\alpha}_n$ and $\mathbf{b}^n \approx \boldsymbol{\beta}^n$. The time scales of the system in Eq. (3.1) can be approximated by the inverse modulo of the eigenvalues of $\mathbf{J}$:

$$\tau_n = \frac{1}{|\lambda_n|}, (n = 1, ..., N), \tag{3.3}$$

where λ_n are the non-zero eigenvalues of $\mathbf{J}$. A zero eigenvalue refers to processes of infinite time scale. A negative (possitive) real part of the eigenvalue indicates a time scale of dissipative (explosive) nature.

Due to its nature, the system is expected to be evolving in different time scales, and it is assumed that the fastest of them, say M, are [i] of dissipative nature (i.e., the components of the system that generate them tend to drive the system towards equilibrium) and [ii] much faster than the rest, then the vector field $\mathbf{g}(\mathbf{y})$ can be decomposed into its fast and slow component:

$$\frac{d\mathbf{y}}{dt} = \mathbf{g}_{\text{fast}}(\mathbf{y}) + \mathbf{g}_{\text{slow}}(\mathbf{y}) = \sum_{r=1}^{M} \mathbf{a}_r f^r + \sum_{s=M+1}^{N} \mathbf{a}_s f^s. \tag{3.4}$$

The fast $\mathbf{g}_{\text{fast}}(\mathbf{y})$ and slow $\mathbf{g}_{\text{slow}}(\mathbf{y})$ components relate to the M fast and $N-M$ slow, respectively, time scales. After the fast dissipative time scales act, they become exhausted and their related modes have no impact on the evolution of the system; i,e. the corresponding amplitudes become negligibly small. Then, a reduced model can be constructed as:

$$f^r \approx 0 \quad (r = 1, \ldots, M), \qquad \frac{d\mathbf{y}}{dt} \approx \sum_{s=M+1}^{N} \mathbf{a}_s f^s. \tag{3.5}$$

The left relation describes an M-dim. system of algebraic equations that define a low dimensional surface in the phase-space, called the *Slow Invariant Manifold (SIM)*, where the system is confined to evolve under the influence of the M fast exhausted time scales. These constraints refer to forming equilibria among fast reactions of the fast variables, and, thus, are characterized by the M fastest timescales, which are inherently dissipative in nature. The right relation is an N-dim. system of ODEs that governs the slow evolution of the system on the *SIM*, the dynamics of which are determined either by the fastest among the $N-M$ slow, dissipative timescales or by explosive timescales, when the latter are present. The explosive timescales, in particular, are the time scales that tend to drive the system away from equilibrium. Such cases arise in all combustion systems [8–11], but also in oscilating biological systems [12], population dynamics [13] and in systems describing cancer evolution [14].

CSP Pointer

Among the several developed algorithmic tools in the context of CSP, the CSP Pointer (Po) is an index that identifies the variables related the most to the a CSP mode. Through iterative analysis, Po assigns a numerical value to each variable and the CSP modes, reflecting its contribution to the fast or slow dynamics of the system [15]. The relation of the the i-th variable to the m-th CSP mode $(m = 1, \ldots, M)$ is calculated as:

$$\mathbf{D}^m = diag\left[\boldsymbol{\alpha}_m \boldsymbol{\beta}^m\right] = \left[\alpha_m^1 \beta_1^m, \alpha_m^2 \beta_2^m, \ldots, \alpha_m^N \beta_N^m\right] \qquad (m = 1, \ldots, M), \tag{3.6}$$

where, due to the orthogonality condition $\boldsymbol{\beta}^i \cdot \boldsymbol{\alpha}_j = \delta_j^i$, the sum of all N elements of $\mathbf{D}^m$ equals unity, i.e. $\sum_{i=1}^N \alpha_m^i \beta_i^m = 1$ [3, 16, 17]. The geometrical interpretation of the Po is presented in [18].

Each variable is associated differently to each CSP mode; A relatively large value of $\alpha_m^i \beta_i^m$ indicates that the i-th variable is strongly associated to the m-th CSP mode and the m-th timescale. For fast exhausted modes, the CSP Pointer identifies the variables related to fast dynamics; i.e. fast variables. In addition, a value of D_i^m close to unity suggests that the i-th variable is in a quasi-steady-state [17].

The CSP Pointer tool is a critical extension of the CSP method, designed to systematically identify fast variables and processes within stiff systems. This allows us to quantitatively identify the dominant fast variables, facilitating the subsequent application of model reduction techniques such as Quasi-Steady State Approximation (QSSA) and Partial Equilibrium Approximation (PEA) [17, 19]. By using the CSP Pointer tool, it is possible to localize the regions in the system where model reduction can be applied most effectively, providing a powerful means of simplifying complex biological or chemical systems while retaining the essential dynamical features.

References

1. Lam SH, Goussis DA. Understanding complex chemical kinetics with computational singular perturbation. In: Symposium (International) on Combustion. vol. 22. Elsevier; 1989. p. 931–941.

2. Lam SH, Coussis D. Conventional asymptotics and computational singular perturbation for simplified kinetics modelling. Reduced Kinetic Mechanisms and Asymptotic Approximations for Methane-Air Flames: A Topical Volume. 2005; p. 227–242.

3. Lam S, Goussis D. The CSP method for simplifying kinetics. International journal of chemical kinetics. 1994;26(4):461–486.

4. Zagaris A, Kaper HG, Kaper TJ. Fast and slow dynamics for the computational singular perturbation method. Multiscale Modeling & Simulation. 2004;2(4):613–638.

5. Kaper HG, Kaper TJ, Zagaris A. Geometry of the computational singular perturbation method. Mathematical modelling of natural phenomena. 2015;10(3):16–30.

6. Chen RTQ, Rubanova Y, Bettencourt J, Duvenaud DK. Neural Ordinary Differential Equations. In: Advances in Neural Information Processing Systems. vol. 31. Curran Associates, Inc.; 2018.Available from: https://proceedings.neurips.cc/paper/2018/hash/69386f6bb1dfed68692a24c8686939b9-Abstract.html.

7. Latrémolière F, Narayanappa S, Vojtěchovský P. Estimating the Jacobian matrix of an unknown multivariate function from sample values by means of a neural network; 2022. Available from: http://arxiv.org/abs/2204.00523.

8. Khalil AT, Manias DM, Tingas EA, Kyritsis DC, Goussis DA. Algorithmic analysis of chemical dynamics of the autoignition of NH3–H2O2/air mixtures. Energies. 2019;12(23):4422.

9. Manias DM, Patsatzis DG, Kyritsis DC, Goussis DA. NH3 vs. CH4 autoignition: A comparison of chemical dynamics. Combustion Theory and Modelling. 2021;25(6):1110–1131.

10. Rabbani S, Manias DM, Kyritsis DC, Goussis DA. Chemical dynamics of the autoignition of near-stoichiometric and rich methanol/air mixtures. Combustion Theory and Modelling. 2022;26(2):289–319.

11. Tingas EA, Gkantonas S, Mastorakos E, Goussis D. The mechanism of propagation of NH3/air and NH3/H2/air laminar premixed flame fronts. International Journal of Hydrogen Energy. 2024;78:1004–1015.

12. Goussis DA, Najm HN. Model reduction and physical understanding of slowly oscillating processes: the circadian cycle. Multiscale Model Sim. 2006;5(4):1297–1332.

13. Manias DM, Patsatzis DG, Goussis DA. Time scale dynamics of COVID-19 pandemic waves: The case of Greece. arXiv preprint arXiv:231207260. 2023;.

14. Patsatzis DG. Algorithmic asymptotic analysis: Extending the arsenal of cancer immunology modeling. Journal of Theoretical Biology. 2022;534:110975.

15. Valorani M, Najm HN, Goussis DA. CSP analysis of a transient flame-vortex interaction: time scales and manifolds. Combust Flame. 2003;134(1-2):35–53.

16. Goussis DA, Lam SH. A study of homogeneous methanol oxidation kinetics using CSP. In: Symposium (International) on Combustion. vol. 24. Elsevier; 1992. p. 113–120.

17. Goussis DA. Quasi steady state and partial equilibrium approximations: their relation and their validity. Combust Theor Model. 2012;16(5):869–926.

18. Manias DM, Goldman RN, Goussis DA. Physical insights from complex multiscale non-linear system dynamics: Identification of fast and slow variables. Communications in Nonlinear Science and Numerical Simulation. 2025;148:108858.

19. Patsatzis DG, Goussis DA. Algorithmic criteria for the validity of quasi-steady state and partial equilibrium models: the Michaelis–Menten reaction mechanism. Journal of Mathematical Biology. 2023;87(2):27.
