# Supplementary material for: Data-driven identification of biological systems using multi-scale analysis
Source: PLoS Comput Biol. 2025 Nov 6;21(11):e1013193. doi: 10.1371/journal.pcbi.1013193 (PMC12611157; doi:10.1371/journal.pcbi.1013193)
Supplement: S5 Appendix — (PDF) [file pcbi.1013193.s005.pdf]

S5 Appendix. CSP diagnostics of the stochastic model of Case 3.

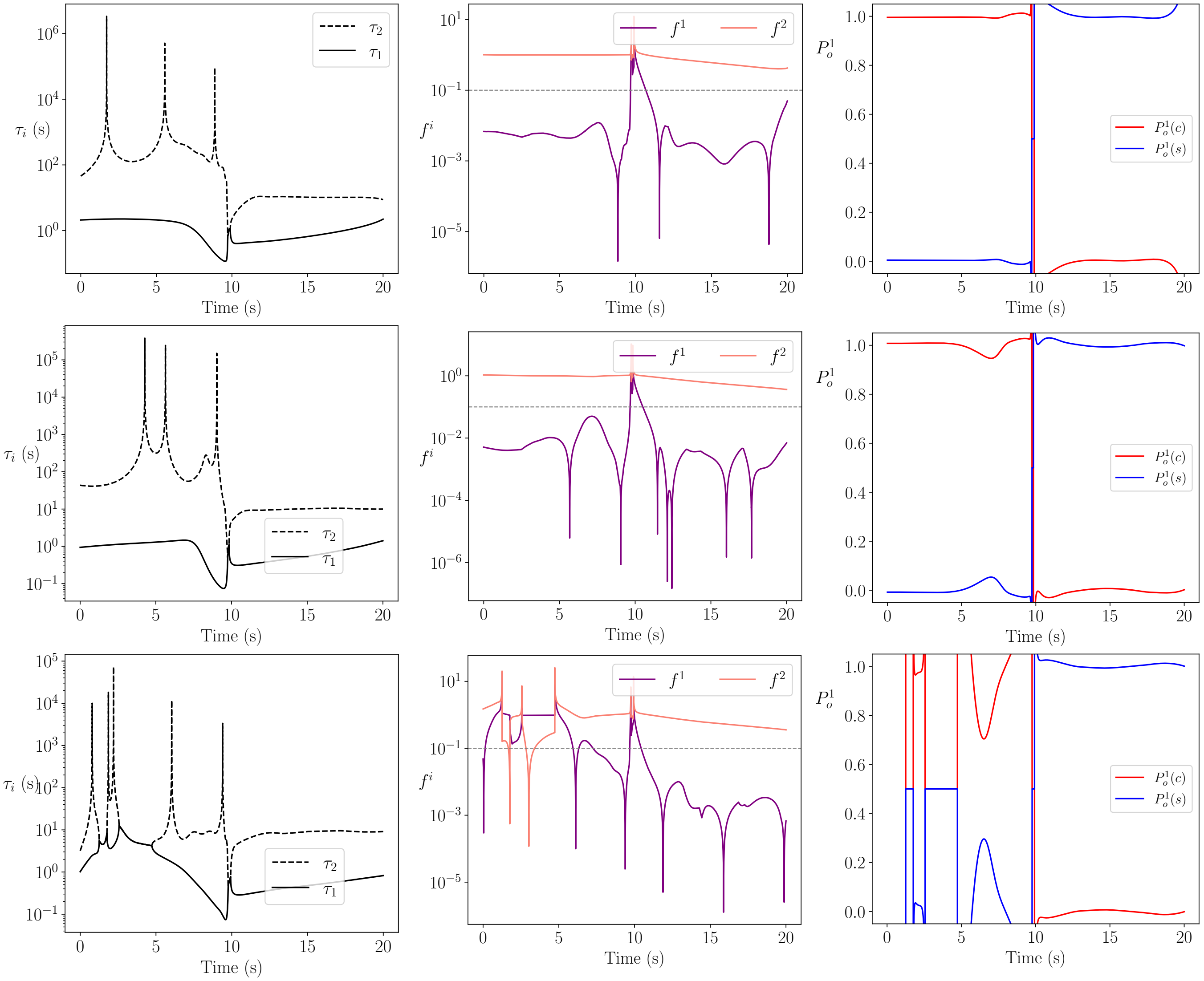

Fig A. CSP Analysis. The evolution in time of the developing time scales (left), the amplitudes of the related CSP modes (center) and the CSP Pointer (right) as they were calculated from the reconstructed vector field from NODE for no noisy data (top), data with additive noise (middle) and data with multiplicative noise (bottom) of Case 3.

Fig A (left column) presents the developing time scales of the stochastic models (middle and bottom columns) in comparison to the reference case of no noise (top row), using a reconstructed and smothened vector field from NODE. In all cases, a time scale gap is evident before and after the transition, with the fastest and driving time scale to be of the same order. The reactivation of the related to the fast mode amplitude (center row) is captures in all cases on the transition, around $t = 10s$.

The CSP Pointer (right column) was able to capture the shift in the dynamics during the reactivation of the fast mode, by identifying the fast variable of each region, indicative of valid sQSSA and rQSSA (Fig 3 and Fig 5 in the main text). These results demonstrate the robustness of our framework in handling noisy data, as the key outcomes remain consistent across different noise levels, enabling a reliable and unified analysis regardless of noise perturbations.
